# Modified Artificial Bee Colony Algorithm-Based Strategy for Brain Tumor Segmentation

**DOI:** 10.1155/2022/5465279

**Published:** 2022-05-11

**Authors:** Priyanka Dahiya, Anil Kumar, Ashok Kumar, Bijan Nahavandi

**Affiliations:** ^1^School of Computing, DIT University, Dehradun, India; ^2^Department of Industrial and Technology Management, Faculty of Management and Economy, Islamic Azad University, Science and Research Branch, Tehran, Iran

## Abstract

Medical image segmentation is a technique for detecting boundaries in a 2D or 3D image automatically or semiautomatically. The enormous range of the medical image is a considerable challenge for image segmentation. Magnetic resonance imaging (MRI) scans to aid in the detection and existence of brain tumors. This approach, however, requires exact delineation of the tumor location inside the brain scan. To solve this, an optimization algorithm will be one of the most successful techniques for distinguishing pixels of interest from the background, but its performance is reliant on the starting values of the centroids. The primary goal of this work is to segment tumor areas within brain MRI images. After converting the gray MRI image to a color image, a multiobjective modified ABC algorithm is utilized to separate the tumor from the brain. The intensity determines the RGB color generated in the image. The simulation results are assessed in terms of performance metrics such as accuracy, precision, specificity, recall, F-measure, and the time in seconds required by the system to segment the tumor from the brain. The performance of the proposed algorithm is computed with other algorithms like the single-objective ABC algorithm and multiobjective ABC algorithm. The results prove that the proposed multiobjective modified ABC algorithm is efficient in analyzing and segmenting the tumor from brain images.

## 1. Introduction

Image segmentation is a branch of digital image processing that focuses on segmenting images based on their features and qualities. Its fundamental purpose is to simplify the image so that it can be analyzed more easily. Medical image segmentation is a technique for automatically or semiautomatically detecting boundaries in a 2D or 3D image. The enormous range of medical images [1] is a substantial challenge for image segmentation. To begin with, significant disparities in human anatomy may be seen. Medical images are created using several methods, including X-rays, MRIs, and others. The segmentation data may then be used to get further diagnostic information. Based on the recovered boundary data, it is possible to do automated organ measurement, cell counting, and simulations. Medical image segmentation is extensively used in image guiding. As a consequence, the advantages and limits of image segmentation are crucial in image-guided surgery [2].

**Figure 1:**
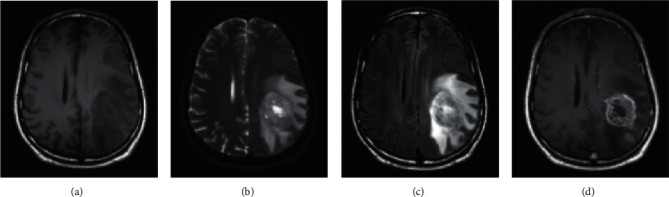
MRI scanning images.

Figure 1 depicts the MRI scanning image from which the tumor is segmented from the brain. Image segmentation is often used in brain MRI analysis to measure and visualize anatomical characteristics, evaluate brain changes, detect diseased regions, plan surgical procedures, and provide image-guided therapy. Traditionally, a brain tumor was thought to be a deadly condition. Even in today's technologically sophisticated society, if the tumor is not found early enough, it might be fatal. Millions of lives may be saved if malignant cells were found early. A brain tumor's form is critical in evaluating its severity. Even if you have all of the parts in place, object detection will be ineffective [3]. Only bounding boxes will be generated, which will not assist us in determining how to shape the cells. Image segmentation algorithms have a significant influence in this case. They allow us to take a more thorough approach to the problem and provide more meaningful results [4].

In this study, to detect the tumor of the brain, image segmentation is accomplished using a multiobjective optimization approach. For this, multiobjective-based modified ABC algorithms are utilized. Multiobjective optimization is a subset of multiple-criteria decision-making that is concerned with problems related to mathematical optimization that involves the simultaneous optimization of numerous objective functions. Multiobjective programming, vector optimization, multicriteria optimization, multiattribute optimization, and Pareto optimization are other names for it. Many fields of study, including engineering, have employed multiobjective optimization to achieve optimal decisions when faced with trade-offs between two or more competing objectives. In many real-world engineering applications, designers must choose between competing goals. The image segmentation problem necessitates dividing a single image into segments, or portions, that contain comparable pixels. Segments are areas of an image that depict the same thing. It is generally termed as an exhaustive partitioning of the image given as input into regions, each of which is homogeneous concerning some image quality of interest.

In this paper, a multiobjective method is developed to improve intercluster distance and hence reduce misclassification. To segregate the tumor from the brain, the modified ABC algorithm-based multiobjective K-means approach is used for the MRI image. The effectiveness algorithm suggested will be determined by measures of [5] confusion matrix-like accuracy, sensitivity, specificity, precision, and recall to compare the efficiency of the proposed algorithm to other algorithms chosen, as well as the time in seconds that a specific algorithm took to segment the tumor from the brain.

### 1.1. Contributions of the Study

The main contributions of the research are as follows:

- To detect the brain tumor by image segmentation using a modified multiobjective Artificial Bee Colony Algorithm
- To analyze the proposed ABC algorithm with other existing algorithms like single-objective ABC and single-objective modified ABC

Furthermore, the article is structured such that Section 2 deals with relevant work, Section 3 outlines the suggested technique, and Section 4 exhibits the findings. Finally, part V concludes the work.

## 2. Literature Review

This section covers the related work of several researchers on MRI segmentation approaches.

### 2.1. K-Means Clustering

The adaptive k-means clustering segmentation method divides the MRI image into segments from which a meaningful extract of the brain tumor may be extracted. Finally, the segmented image is classified using a Support Vector Machine classifier. This classifier determines the kind of tumor. When the linear kernel function of three SVM classifier kernel functions is compared, it yields a more accurate result [6]. In 2021, Sangeeta et al. reported on an effective image divisions approach based on K-implies bunching. To identify brain tumors precisely, sifting, thresholding, Otsu binarization [7], and segmentation stages are utilized. A median channel is a filtering technique used to remove disturbances from an MRI image [8]. The recommended procedure may employ the K technique grouping for image segmentation by utilizing the least handling instance. The proposed approach has been approved on the BRATS 2015 data set [9].

Authors have proposed an ABC-based method for tackling limited optimization problems that use Deb's principles as a selection mechanism [10]. Their suggested technique performed well when it came to handling difficult numerical optimization tasks [11]. Inspired by PSO, Zhu and Kwong proposed a gbest-guided ABC [11]. This algorithm employs the global best in the search process and outperforms ABC in terms of exploitation. Banharnsakun, Achalakul, and Sirinaovakul recommended that the swarm discuss the most practicable solutions discovered so far [12]. They also presented an adaptive search radius adjustment technique.

The Artificial Bee Colony (ABC) and Fuzzy-C Means (FCM) algorithms are used by Neeraja Menon and Rohit Ramakrishnan [13] to present a rapid MRI Brain Image segmentation approach. A threshold estimate is used to find a value in a continuous grayscale interval. The ABC algorithm is used to find the appropriate threshold value. The original picture is deconstructed using discrete wavelet transformations to provide an effective fitness function for the ABC method. After that, a filtered picture rebuilt with low-frequency components is created by applying noise reduction to the approximation image. For clustering the segmented picture, the FCM method is applied, which aids in the identification of the brain tumor.

3D Magnetic Resonance Imaging (3D-MRI) segmentation of brain tumors is an essential tool for gathering information needed for diagnosis and disease treatment planning. One of the key obstacles in tumor segmentation is variation in tumor size, structure, and shape, and picking the starting contour plays a vital role in lowering segmentation error and the number of iterations in the level set technique. To solve this problem, Khalil et al. [14] propose a two-step dragonfly algorithm (DA) clustering approach to reliably extract initial contour points in their study. In the preprocessing stage, the brain is removed from the skull, then tumor edges are extracted using the two-step DA and utilized as a starting contour for the MRI sequence. Finally, a level set segmentation algorithm is used to recover the tumor area from all volume slices. The findings of applying the suggested methodology to 3D-MRI images from the multimodal brain tumor segmentation challenge (BRATS) 2017 data set reveal that the proposed method is comparable to state-of-the-art techniques for brain tumor segmentation.

Brain cancer must be discovered sooner so that the treatment procedure may be carried out more accurately and patients' lives can be extended. Machine learning algorithms may be used to aid brain cancer prediction based on the kind of tumor using microarray data. A multiclass classification issue may be used to describe this situation. [15]. As a feature selection approach, Multiple Multiclass Artificial Bee Colony (MMABC) was used, and as a classification method, Support Vector Machine (SVM) was used. SVM may generate accurate and robust classification results, and the ABC approach has proven useful in tackling optimization issues with the large dimensionality. The information was gathered from the Broad Institute. There are 7129 characteristics and 42 samples in the data. According to the results of the experiment, the accuracy of Multiple SVM employing a feature selection-based MMABC approach achieved 95.24 percent in the use of 300 best features, which is somewhat higher than the accuracy of SVM without feature selection.

Aside from numerical function optimization, there are several research investigations on ABC applications in domains including data clustering [16, 17] and solar system design [18].

## 3. Methodology

A modified multiobjective ABC algorithm optimization strategy is proposed after evaluating the data and using the suggested algorithms to segment the images to find the centroid of each cluster, and the model is compared to other optimization strategies to show its efficacy.

The suggested algorithm's process is as follows.

### 3.1. Data Set

The BTS (Brain Tumor Segmentation) data set, which is made up of images from magnetic resonance imaging (MRI) scans, was used in the research. A total of 50 DICOM files comprising brain images were extracted from the data set and statistically analyzed. For tumor segmentation, a single image is used from the data. With each of the three methods, the same image is utilized for analysis.

### 3.2. Data Preprocessing

The initial step in doing analysis is to load the data and prepare it so that it can be analyzed properly. The uploaded image was determined to be in monochrome format; however, grayscale photographs are difficult to detect to identify cancers; thus, we converted the image to color. The application of color labeling allowed for more precise tumor site designation. The loaded image is subjected to image enhancement and image contrast techniques. The goal of image enhancement is to improve the image either subjectively or objectively. Intensity adjustment is an image-enhancing technique that remaps an image's intensity values to a new range. The difference between the image's peak and lowest intensity values is calculated using image contrast.

Preprocessing MRI images with a color band to increase their quality and make the tumor zone segmentation process easier.

### 3.3. Image Segmentation

This research employs three segmentation techniques. They include single-objective K-means based on ABC, single-objective K-means based on modified ABC algorithms, and multiobjective K-means based on modified ABC algorithms. A grayscale image is difficult to analyze, as stated above in the preparation section; thus, the image is transformed into a color image for easier analysis. The image is then segmented using the above-stated methods, and a comparative study is carried out to validate their strengths in tumor segmentation.

#### 3.3.1. Segmentation of MRI Image Using K-Means

Before pattern identification, feature extraction, and image reduction, segmentation is often employed as a preprocessing step. There are various methods available; however, the K-Means clustering technique is one of the most often utilized. The K-Means clustering methodology is an unsupervised method for distinguishing the region of interest, which is a tumor, from the surrounding area. It divides the input three-dimensional MRI image into two clusters or sections based on the two centroids. The goal is to detect and group the background pixels and tumor pixels.

K-means organizes three-dimensional data vectors into predetermined number clusters (background, tumor). The centroid vector of each cluster is started with an arbitrary vector [19]. The mean of the connected data vectors is reflected in each centroid vector. When a data vector is clustered, it is shown by pixel in the image, following the clustering of all pixels. This technique is repeated until there are no substantial changes in the cluster mean.

As a result, the K-means clustering algorithm may be described as

(i) Initiate the cluster means of the background and tumor pixels at random
(ii) Repeat
  (1) Using Euclidean distance, assign each pixel in the MRI image
  (2) Determine the means of each cluster using
    (1) $$\begin{array}{c} S_{i}=\frac{1}{m_{i}}\sum_{\forall Z_{w}\in C_{i}}Z_{w}, \end{array}$$
  where *m*_*i*_ = total pixels from cluster *i* and *C*_*i*_ = *C* represents the pixel subset that constitutes cluster *i* up to the point at which a halting requirement is met.

In this study, K-means and modified ABC algorithms are evaluated using a fixed number of iterations (*t*_max_) as the stopping condition. This provides for a fair comparison of the performance of the two methods. If there are no significant changes in the mean vectors, the clustering process may be terminated, which is an alternative technique [20]. Because of their high computational complexity, K-means algorithms are computationally expensive owing to their repeating nature.

#### 3.3.2. Modified Algorithm-Based Multiobjective MRI Image Segmentation

The term parameter selection refers to genetic algorithms that are used to change the parameters of an existing image segmentation technique to improve its output, whereas pixel-level segmentation refers to genetic algorithms that are used to improve the output of an existing image segmentation technique. Modified ABC algorithms are utilized at the pixel level to tag regions. The first strategy is utilized more often in the bulk of image segmentation algorithms.

**Figure 2:**
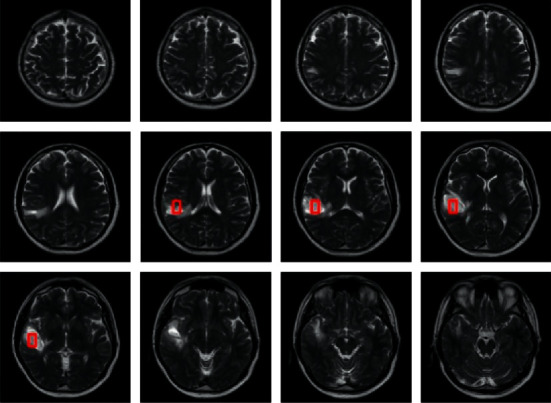
Brain tumor detection.

Figure 2 depicts the identification of brain tumors using modified ABC algorithm-based image clustering, a gbest GA image clustering technique, in which each particle's quality is assessed using

(2) $$\begin{array}{c} f\left(x_{j},Z_{j}\right)=y_{1}{\bar{d}}_{\max}\left(Z_{j},x_{j}\right)+y_{2}\left(Z_{\max}-d_{\min}\left(x_{j}\right)\right), \end{array}$$

where *Z*_max_ is the image set's maximum pixel value. The user-defined constants *y*_1_*y*_2_ are used for weighing the contribution of individual subobjectives.

(3) $$\begin{array}{c} {\bar{d}}_{\max}\left(Z_{j},x_{j}\right)=\underset{k=1\ldots M}{\max}\left\{\sum_{\forall Z_{b}\in E_{jk}}\frac{D\left(Z_{b},h_{jk}\right)}{\left|E_{jk}\right|}\right.. \end{array}$$

The largest average Euclidean distance between particles and their clusters is *d* and ${\bar{d}}_{\max}$, which is equal to the sum of all the clusters' greatest distances shown by equation the following equation:

(4) $$\begin{array}{c} d_{\min}\left(x_{j}\right)=\underset{\forall k_{1},k_{2},k_{1}\neq k_{2}}{\min}\left\{d\left(h_{jk_{1}},h_{jk_{2}}\right)\right\}. \end{array}$$

In the case of a single objective-based algorithm, the first half of equation (2) is only used for segmentation; however, it has a propensity to misclassify. This may be avoided by incorporating the second portion, which encourages the increase of intercluster distance, or the distance between centroids, and therefore reduces the number of misclassifications. According to the fitness function's derivation, a small value *f*(*x*_*j*_, *Z*_*j*_) suggests compact and well-separated clusters.

**Figure 3:**
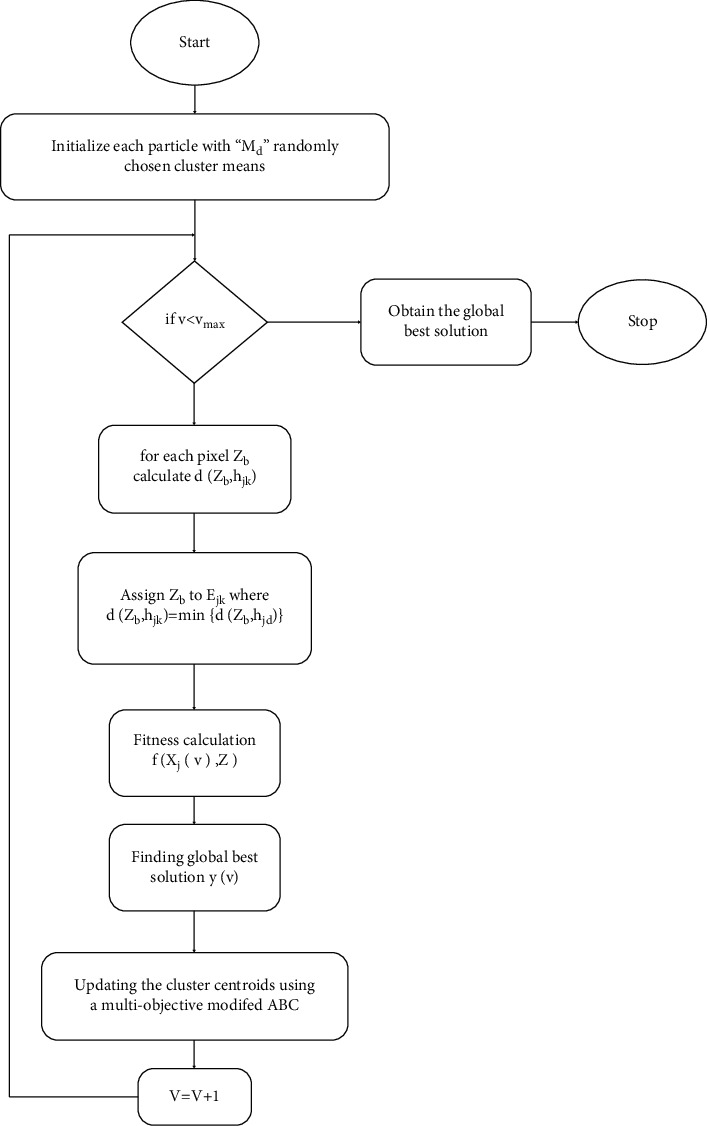
Algorithm flow chart.

As a consequence, the fitness function is a problem with several objectives. The majority of multiobjective problem-solving approaches have been created for evolutionary algorithms. Lately, multiobjective optimization approaches based on the genetic algorithm have been created. A simple method is used to handle various aims as our purpose is to illustrate the application and usability of an algorithm for image clustering. The subobjectives are given different priorities by suitably initializing the values of *w*_1_ and *w*_2_. The flow chart of the modified ABC algorithm-based image clustering algorithm is depicted in Figure 3 [21].

### 3.4. Basic Artificial Bee Colony Algorithm

Among the most current swarm-based algorithms is the Artificial Bee Colony (ABC) algorithm. ABC replicates a honeybee swarm's clever foraging activity. The honey bee colony model in the ABC algorithm has three types of bees: worker, spectator, and scout [20]. ABC starts by randomly distributing an initial population of SN solutions using the following equation:

(5) $$\begin{array}{c} x_{i}^{j}=x_{\min}^{j}+r\text{ and }\left[0,1\right]\left(x_{\max}^{j}-x_{\min}^{j}\right), \end{array}$$

where *i* = 1,…, SN and *j* = 1,…, *D*. *D* is the number of optimization parameters or *D* is the dimension. The parameter bounds are *x*_min_ and *x*_max._

Each engaged bee creates a new potential solution *V*_*i*_ in the vicinity of its present location by the following equation:

(6) $$\begin{array}{c} v_{i}^{j}=x_{i}^{j}+\phi_{i}^{j}\left(x_{i}^{j}-x_{k}^{j}\right). \end{array}$$

Onlooker bees begin to work once employed bees have finished their assignments. An observer bee selects a food source based on its nectar value, which is calculated using the following equation:

(7) $$\begin{array}{c} P_{i}=\frac{{\text{fit}}_{i}}{\sum_{n=1}^{SN}{\text{fit}}_{n}}, \end{array}$$

where fit_*i*_ is computed by the following equation:

(8) $$\begin{array}{c} fit_{i}=\left\{\begin{array}{c} \frac{1}{\left(1+{\text{fitness}}_{i}\right)},\;\text{if }\left({\text{fitness}}_{i}\geq 0\right)\\ 1+abs\left({\text{fitness}}_{i}\right),\;\text{if }\left({\text{fitness}}_{i}<0\right) \end{array}\right\}. \end{array}$$

That fitness_*i*_ is the solution's nectar (fitness) value. It's worth noting that onlooker bees employ (6) to produce new solution candidates as well.

After a certain number of repetitions, if a food source does not improve (called Limit), the food source is deemed abandoned. In such a scenario, a scout bee is sent to search for a new food source for replacing the one that has been abandoned. This new location was created with the help of (5).

### 3.5. Proposed Algorithm

The proposed algorithm in this paper is modified ABC which was taken from the [22]. The modifications made were, first, the whole scout bee technique and, second, the process of establishing new neighbors for both observer and employed bees.

## 4. Result

MATLAB 2020a was used to implement this model. For analysis, a sample of four MRI scan test images was selected, as indicated in Tables 1to 3. The suggested and comparative methods are evaluated using the same four test images. These tables show how the test scans changed over time, from the original image to the enhanced image, gray-labeled image, color-mapped image, and tumor-segmented image. The image segmentation procedure using multiobjective modified ABC is shown in Table 1.

Table 2 shows the image segmentation process using a single-objective modified ABC algorithm. The image segmentation process utilizing the single-objective ABC is shown in Table 3. The table's final column depicts the segmentation of the tumor from the brain utilizing certain algorithms. The suggested technique may be seen in the segmented scans of the three tables below. Single-objective modified ABC and single objective ABC methods give less effective outcomes for test images than multiobjective modified ABC algorithms.

To assess the effectiveness of the algorithms, performance parameters [1], namely, precision, accuracy, specificity, sensitivity, and F-measure are evaluated. The following formulae are used to determine these parameters:

(9) $$\begin{array}{c} \text{Accuracy}=\frac{TP+TN}{TP+TN+FP+FN},\\ \text{Sensitivity}=\frac{TP}{TP+FN},\\ \text{Specificity}=\frac{TN}{TN+FP},\\ \text{Precision}=\frac{TP}{TP+FP},\\ F-\text{measure}=2*\frac{\text{Precision}*\text{Recall}}{\text{Precision}+\text{Recall}}, \end{array}$$

where *TP*= true positive, *TN*= true negative, *FP*= false positive, and *FN*= false negative.

Initially, a data set of ten samples was used for the experiment, and the performance parameters were examined. The parameters and time required by the system to segment the image have been analyzed.  Table 4 summarizes the values of the multiobjective modified ABC algorithm.  Table 5 summarizes the values of the single-objective modified ABC algorithm.

Table 6 summarizes the values of performance parameters and time in seconds for segmenting a single-objective ABC algorithm. It shows the precision, sensitivity, F-measure, accuracy, specificity, and time (sec) in respect of the number of image samples.

**Figure 4:**
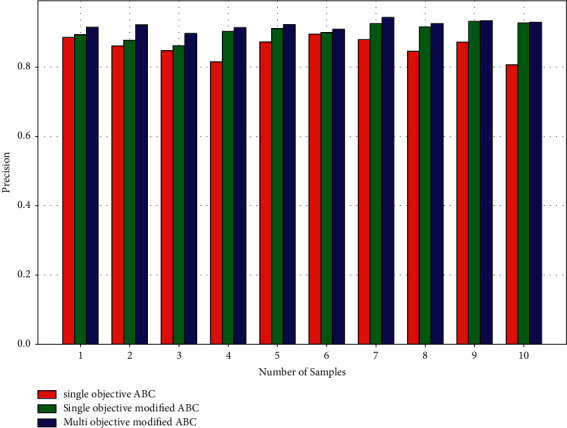
Performance parameters—precision.

Figure 4 depicts a precision study of three methods: the average accuracy of the single-objective ABC Algorithm is 0.85, the single-objective modified ABC Algorithm is 0.90, and the multiobjective modified ABC Algorithm is 0.92. Single-objective ABC has 0.05 less accuracy than single-objective modified ABC while raising the objective from single to multi has boosted precision by 0.02. When compared to other algorithms, the multiobjective modified ABC algorithm produces excellent results in tumor segmentation.

**Figure 5:**
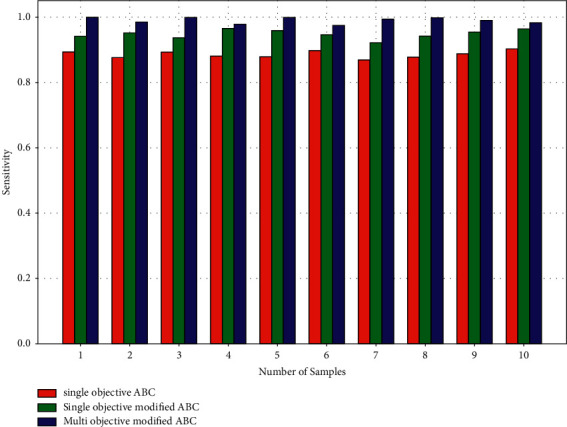
Performance parameters—sensitivity.

Sensitivity refers to the recall levels of the findings utilized for segmentation.  Figure 5 shows that the single-objective ABC algorithm has lower sensitivity than the single- and multiobjective modified ABC algorithms, while the multiobjective modified ABC method has greater sensitivity than the single-objective modified ABC algorithm. The single-objective ABC average is 0.980, the single-objective modified ABC average is 0.983, and the multiobjective modified ABC average is 0.989. When single objective ABC is joined with single-objective modified ABC, an increase of 0.003 is seen, and a 0.006 increment in multiobjective modified ABC when compared to single-objective modified ABC is seen. It can be seen that the algorithm sensitivity values have very minimal increases. Sensitivity values are included in the suggested algorithms.

**Figure 6:**
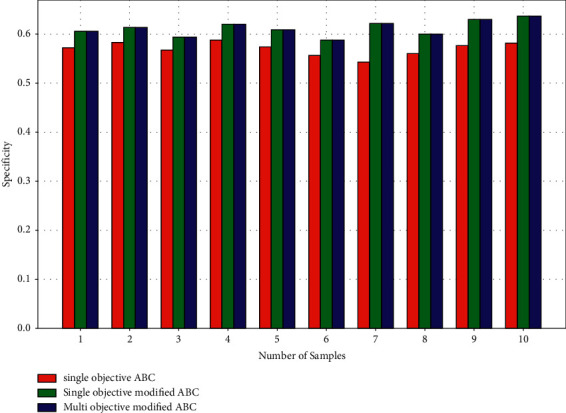
Performance parameters—specificity.

The specificity values of the three techniques are shown in Figure 6. It shows that the specificity of the three algorithms is near to each other. However, when the averages are determined, single-objective ABC has 0.57, single-objective modified ABC has 0.61, and suggested method multiobjective modified ABC has 0.64. When compared to single-objective modified ABC, single-objective modified ABC has a specificity of 0.04 while multiobjective modified ABC has a specificity of 0.03. As a result, the suggested method has a higher specificity than prior techniques.

**Figure 7:**
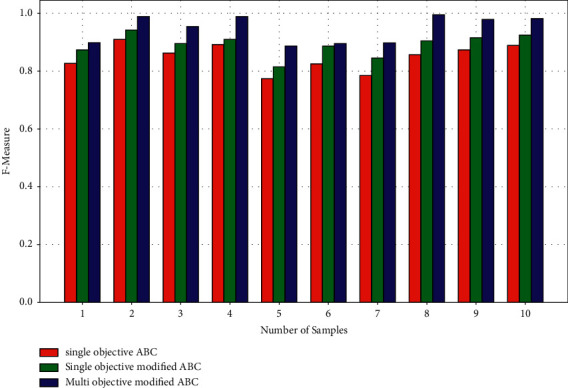
Performance parameters—F-measure.

Figure 7 shows the higher F-measure than the other two algorithms. The average F-measure for single-objective ABC is 0.84, 0.89 for single-objective modified ABC, and 0.94 for multiobjective modified ABC. As a result, when compared to single-objective modified ABC, the efficacy of the single-objective ABC algorithm is less than 0.5 F-measure. Furthermore, single-objective modified ABC has 0.5 less F-measure than multiobjective modified ABC. As a consequence, the suggested approach outperforms the single-objective modified ABC algorithm in terms of tumor segmentation from an MRI image.

**Figure 8:**
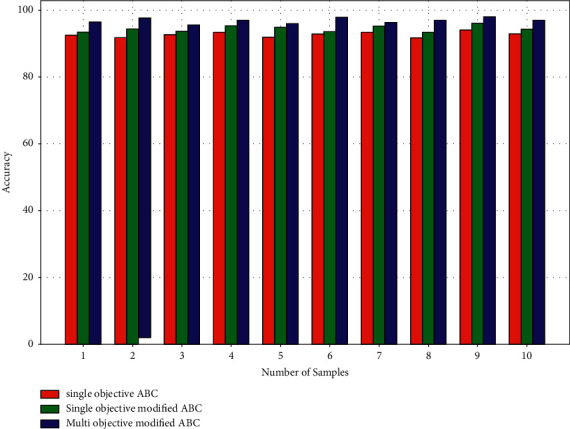
Performance parameters—accuracy.

Figure 8 compares the accuracy of the algorithm findings for ten test samples. The average accuracy of the single-objective ABC algorithm is 92.61 percent, 94.30 percent for the single-objective modified ABC method, and 96.72 percent for the multiobjective modified ABC algorithm. As a result, the multiobjective modified ABC algorithm is more accurate in recognizing tumor regions. When single-objective ABC is combined with single-objective modified ABC, accuracy increases by 2%, and accuracy increases by 2% when the goal is changed from single to multiobjective.

**Figure 9:**
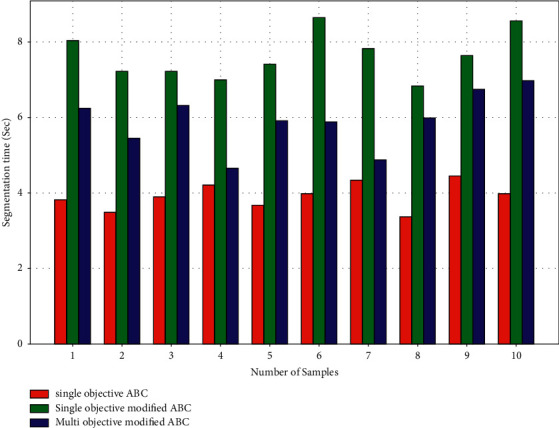
Performance parameters—segmentation time.

Figure 9 compares the time required by algorithms to segment the tumor. The single-objective ABC algorithm takes 3.9 seconds on average to segment the tumor, 7.6 seconds for single objective modified ABC, and 5.9 seconds for multiobjective modified ABC. When paired with the single-objective ABC method, the multiobjective modified ABC optimization algorithm takes 1.7 seconds less time than the single-objective modified ABC algorithm. As a consequence, among the offered image segmentation methodologies, the suggested multiobjective modified ABC algorithm outperforms the other comparison algorithms.

## 5. Conclusion

This implementation was carried out using MATLAB 2020a. The BTS (Brain Tumor Segmentation) data set was employed in the study, which comprises scans from magnetic resonance imaging (MRI) scans. The data set had 50 DICOM files with brain scans that were statistically evaluated. A random sample of four images is chosen from the data set and evaluated using the suggested technique. K-means is based on multiobjective modified ABC algorithm and comparison with single-objective modified ABC algorithm and single-objective ABC algorithm. A grayscale image is selected from the data set and converted to a color image. Image enhancement and image contrast methods are used to improve the image and determine the highest and lowest intensity values. Color labeling is applied to the image to distinguish the tumor from the native brain, and the image is then segmented to emphasize the tumor pixels. The suggested method's performance is assessed using performance parameters and the time required by the specific algorithm to segment the tumor from the brain. According to the above tables and graphs, it is depicted that the performance parameters of the proposed model and the existing algorithms are evaluated. The precision, sensitivity, F-measure, accuracy, specificity, and time in sec are evaluated. 10 sample images are chosen for the analysis, and the proposed model is implemented along with the existing algorithms [23]. The highest precision value for all the proposed algorithm samples is 0.9432, and the least precision is 0.8976. The highest sensitivity value is 0.9998, and the lowest value is 0.9734. The highest F-measure value of the model is 0.9965, and the lowest F-measure is 0.8872. The accuracy is 97.87, and the minimum accuracy is 95.33. Specificity is high at 0.6376 and low at 0.6209. The lowest processing time of the model is 4.65, and the highest is 6.99. Hence, the suggested algorithm's average accuracy is 96 percent, its average sensitivity is 0.989, its average specificity is 0.64, its average F-measure is 0.94, its average precision is 0.92, and its average time for segmenting the tumor is 5.9 seconds. All of these performance parameter assessments show that the suggested method of multiobjective modified ABC algorithm produces effective results when compared to existing algorithms such as single-objective modified ABC algorithm-based K-means and single-objective ABC Algorithm. As a consequence, the suggested method performs well in recognizing and segmenting the tumor from the brain in MRI images.

## Figures and Tables

**Table 1:** Image segmentation using multiobjective modified ABC algorithm.

Test images	Original	Enhanced	Gray	Color labeled	Segmented image
Test image 1	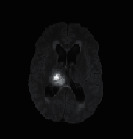	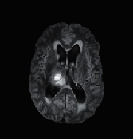	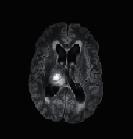	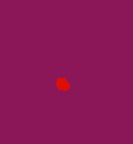	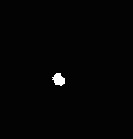
Test image 2	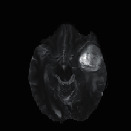	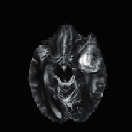	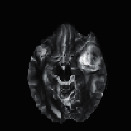	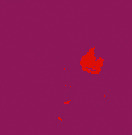	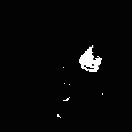
Test image 3	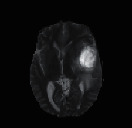	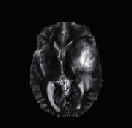	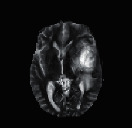	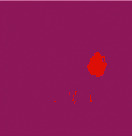	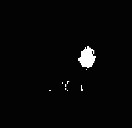
Test image 4	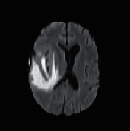	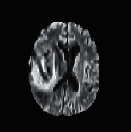	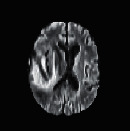	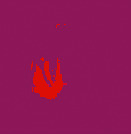	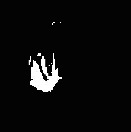

**Table 2:** Image segmentation process using single-objective modified ABC algorithm.

Test images	Original	Enhanced	Gray	Color labeled	Segmented image
Test image 1	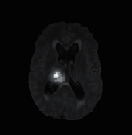	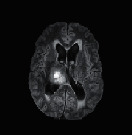	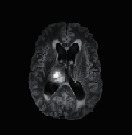	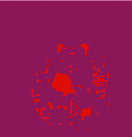	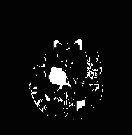
Test image 2	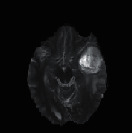	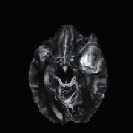	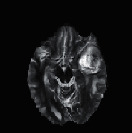	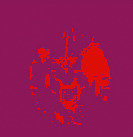	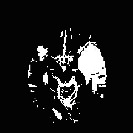
Test image 3	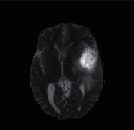	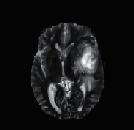	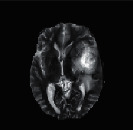	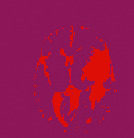	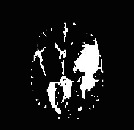
Test image 4	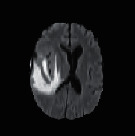	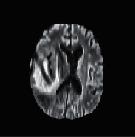	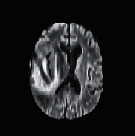	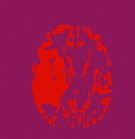	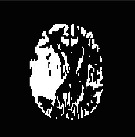

**Table 3:** Image segmentation using single-objective ABC.

Test images	Original	Enhanced	Gray	Color labeled	Segmented image
Test image 1	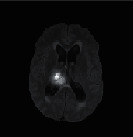	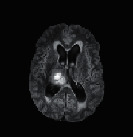	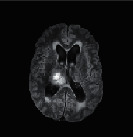	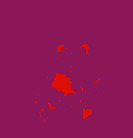	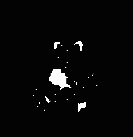
Test image 2	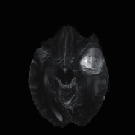	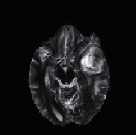	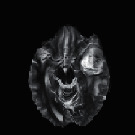	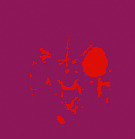	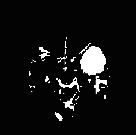
Test image 3	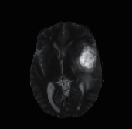	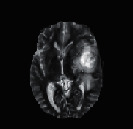	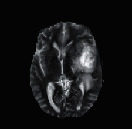	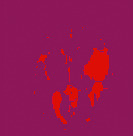	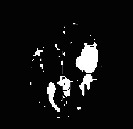
Test image 4	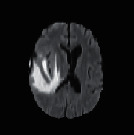	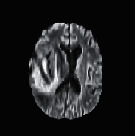	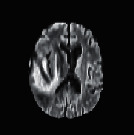	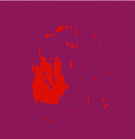	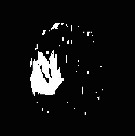

**Table 4:** Performance parameters and time (sec) of multiobjective modified ABC algorithm.

Samples	Precision	Sensitivity	F-measure	Accuracy	Specificity	Time (sec)
1	0.9154	0.9998	0.8987	96.43	0.6059	6.24
2	0.9212	0.9854	0.9874	97.43	0.6132	5.45
3	0.8976	0.9995	0.9543	95.33	0.5943	6.32
4	0.9123	0.9775	0.9872	96.78	0.6202	4.65
5	0.9222	0.9987	0.8872	95.87	0.6089	5.9
6	0.908	0.9734	0.8973	97.87	0.5885	5.89
7	0.9432	0.9934	0.8972	96.04	0.6209	4.89
8	0.9231	0.9991	0.9965	96.87	0.6011	5.98
9	0.9342	0.9886	0.9763	97.77	0.6289	6.76
10	0.9298	0.9787	0.9832	96.87	0.6376	6.99

**Table 5:** Performance parameters and time (sec) of single-objective modified ABC.

Samples	Precision	Sensitivity	F-measure	Accuracy	Specificity	Time (sec)
1	0.8934	0.9421	0.8751	93.26	0.6059	8.04
2	0.8773	0.9512	0.9424	94.32	0.6132	7.23
3	0.8609	0.9359	0.8961	93.43	0.5943	7.20
4	0.9023	0.9645	0.9118	95.12	0.6202	6.99
5	0.9112	0.9574	0.8143	94.64	0.6089	7.42
6	0.8996	0.9465	0.8875	93.62	0.5885	8.65
7	0.9247	0.9224	0.8465	95.12	0.6209	7.85
8	0.9145	0.9471	0.9053	93.46	0.6011	6.84
9	0.9319	0.9565	0.9162	95.73	0.6289	7.65
10	0.9272	0.9623	0.9253	94.33	0.6376	8.56

**Table 6:** Performance parameters and time (sec) of single-objective ABC.

Samples	Precision	Sensitivity	F-measure	Accuracy	Specificity	Time (sec)
1	0.8848	0.8915	0.8271	92.34	0.5721	3.81
2	0.8612	0.8761	0.9124	91.59	0.5832	3.49
3	0.8459	0.8929	0.8626	92.63	0.5674	3.9
4	0.8139	0.8817	0.8921	93.22	0.5874	4.21
5	0.8712	0.8787	0.7731	91.84	0.5743	3.67
6	0.8956	0.8965	0.8251	92.92	0.5575	3.99
7	0.8777	0.8682	0.7827	93.31	0.5434	4.34
8	0.845	0.8775	0.8565	91.54	0.5598	3.38
9	0.8719	0.8843	0.8736	94.02	0.5774	4.46
10	0.8064	0.9023	0.8906	92.77	0.5823	3.97

## Data Availability

The data that support the findings of this study are available from the corresponding author upon reasonable request.
